# The impact of historical redlining on neurosurgeon distribution and reimbursement in modern neighborhoods

**DOI:** 10.3389/fpubh.2024.1364323

**Published:** 2024-05-07

**Authors:** Jean-Luc K. Kabangu, John E. Dugan, Benson Joseph, Amanda Hernandez, Takara Newsome-Cuby, Danny Fowler, Momodou G. Bah, Lane Fry, Sonia V. Eden

**Affiliations:** ^1^Department of Neurological Surgery, University of Kansas Medical Center, Kansas City, KS, United States; ^2^University of Tennessee Health Science Center College of Medicine, Memphis, TN, United States; ^3^Department of Surgery, University of Tennessee Health Science Center, Memphis, TN, United States; ^4^University of Michigan Medical School, Ann Arbor, MI, United States; ^5^Kansas City University College of Osteopathic Medicine, Kansas City, MO, United States; ^6^New York Institute of Technology College of Osteopathic Medicine at Arkansas State University, Jonesboro, AR, United States; ^7^Michigan State University College of Human Medicine, East Lansing, MI, United States; ^8^University of Kansas School of Medicine, Kansas City, KS, United States; ^9^Department of Neurosurgery, University of Tennessee Health Science Center, Memphis, TN, United States; ^10^Semmes-Murphey Neurologic and Spine Institute, Memphis, TN, United States

**Keywords:** structural racism, redlining, neurosurgery, access, healthcare

## Abstract

**Background:**

This study examines the lasting impact of historical redlining on contemporary neurosurgical care access, highlighting the need for equitable healthcare in historically marginalized communities.

**Objective:**

To investigate how redlining affects neurosurgeon distribution and reimbursement in U.S. neighborhoods, analyzing implications for healthcare access.

**Methods:**

An observational study was conducted using data from the Center for Medicare and Medicaid Services (CMS) National File, Home Owner’s Loan Corporation (HOLC) neighborhood grades, and demographic data to evaluate neurosurgical representation across 91 U.S. cities, categorized by HOLC Grades (A, B, C, D) and gentrification status.

**Results:**

Of the 257 neighborhoods, Grade A, B, C, and D neighborhoods comprised 5.40%, 18.80%, 45.8%, and 30.0% of the sample, respectively. Grade A, B, and C neighborhoods had more White and Asian residents and less Black residents compared to Grade D neighborhoods (*p* < 0.001). HOLC Grade A (OR = 4.37, 95%CI: 2.08, 9.16, *p* < 0.001), B (OR = 1.99, 95%CI: 1.18, 3.38, *p* = 0.011), and C (OR = 2.37, 95%CI: 1.57, 3.59, *p* < 0.001) neighborhoods were associated with a higher representation of neurosurgeons compared to Grade D neighborhoods. Reimbursement disparities were also apparent: neurosurgeons practicing in HOLC Grade D neighborhoods received significantly lower reimbursements than those in Grade A neighborhoods ($109,163.77 vs. $142,999.88, *p* < 0.001), Grade B neighborhoods ($109,163.77 vs. $131,459.02, *p* < 0.001), and Grade C neighborhoods ($109,163.77 vs. $129,070.733, *p* < 0.001).

**Conclusion:**

Historical redlining continues to shape access to highly specialized healthcare such as neurosurgery. Efforts to address these disparities must consider historical context and strive to achieve more equitable access to specialized care.

## Introduction

The enduring legacy of structural racism continues to cast a shadow over American society, with the current healthcare landscape of the United States (U.S.) being no exception. Within healthcare, structural racism manifests through disparities in access to care, quality of care, and health outcomes, creating a stark divide that disproportionately affects marginalized communities (1–3). Among the many factors contributing to these disparities, historical practices such as redlining, deeply rooted in the nation’s history, stand out as particularly pernicious (1–4).

Structural racism refers to systematic and institutionalized discrimination against certain racial and ethnic groups, resulting in unequal access to opportunities and resources (5). Redlining serves as a glaring example of such discriminatory practices. Instituted in the 1930s by the Homeowners’ Loan Corporation (HOLC) as part of federal housing policy, redlining assigned neighborhoods a letter grade based on the perceived creditworthiness of inhabitants (6, 7). These grades were heavily influenced by the area’s racial composition, with predominantly Black neighborhoods often receiving the lowest classification– Grade D.

Under the guise of assessing financial risk, redlining effectively segregated communities along racial lines and denied qualified residents in Black neighborhoods access to mortgages and other financial services, perpetuating economic and social inequalities (8). Despite being outlawed by the Fair Housing Act of 1968, lasting consequences of redlining continue to reverberate through American neighborhoods, having a profound effect in exacerbating healthcare disparities (9).

Healthcare disparities rooted in historical injustices have been the subject of extensive research, and their consequences extend across a wide spectrum of medical specialties (1–4). Neurosurgery is specialized and resource intensive, disparities in access may reflect broader disparities of infrastructure and resources. There are limited studies on the influence of redlining on access to Neurosurgery. Neurosurgery offers an illuminating lens through which to examine the ramifications of historical redlining, as it requires specialized infrastructure, advanced equipment, and highly trained professionals, making it particularly sensitive to disparities in healthcare access. By investigating the distribution of neurosurgical services in areas historically affected by redlining, we can elucidate how systemic disparities persist, impacting access to critical healthcare resources. The objective of this study is to investigate the lasting impact of redlining on present-day geographic distribution of neurosurgeons in the U.S. at the neighborhood level and to elucidate its effects on Medicare reimbursement patterns. We posit that redlining, by perpetuating socioeconomic disparities and residential segregation, has had an enduring impact on the availability of specialized medical care in marginalized communities.

## Methods

Institutional Review Board approval was not sought for this study, as no individuals were directly involved or identifiable in the research process, and we utilized publicly accessible data. All study procedures adhere to the Strengthening the Reporting of Observational Studies in Epidemiology (STROBE) guidelines.

### Data source

We obtained data from the Center for Medicare and Medicaid Services (CMS) National Downloadable File (NDF) to identify neurosurgeons practicing in the U.S. from 2020 to September 2023, utilizing Zone Improvement Plan (ZIP) codes. The NDF datasets offer comprehensive information about healthcare providers enrolled with Medicare.

**Figure 1:**
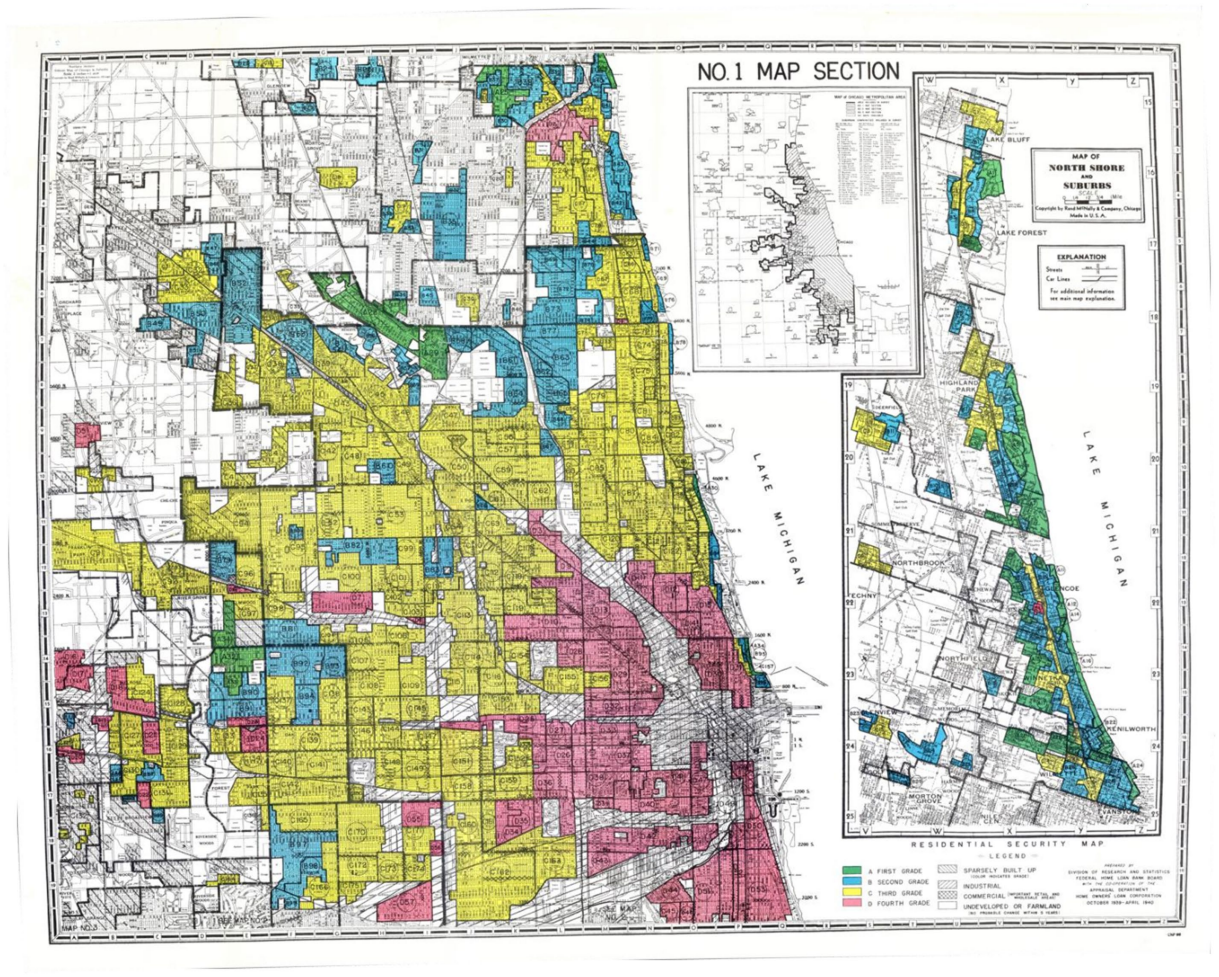
Homeowners’ Loan Corporation (HOLC) Map of Chicago: Displays a (HOLC) map of Chicago. A historic document illustrating the city’s division into distinct residential security grades. The color scheme denotes this division, with green representing Grade A neighborhoods, the most desirable areas for real estate investment. Blue represents Grade B, yellow represents Grade C, and red signifies Grade D neighborhoods, which denotes the redlined areas indicating high risk and disinvestment. Scans of historic HOLC maps are accessible in the public domain, sourced from the National Archives, offering valuable insights into historical urban development practices and their impact on communities.

The deliberate choice of the years 2020–2023 stems from our intention to align this data with demographic information from the latest census, conducted in 2020. This alignment allows for a robust examination of the relationship between neurosurgical practice patterns and demographic factors. By integrating the most recent census data, we ensure that our analysis accurately reflects current population demographics, enhancing the relevance and reliability of our findings. While we acknowledge that conducting the study during the COVID-19 pandemic poses a potential limitation, it’s noteworthy that various publications have indicated improved burnout and satisfaction among neurosurgery applicants, residents, and attending physicians during this time, leading to reduced turnover (10–12). This evidence suggests that the pandemic’s influence on neurosurgical practice dynamics may not significantly impact some of our study’s outcomes. Additionally, we acquired neurosurgeon claims data from the Medicare for Physicians and Other Practitioners by Provider file for the years 2020 and 2021. This dataset, organized by ZIP code, provides comprehensive information on total annual claims submitted by providers and annual payments received. It encompasses details on services and procedures administered to Original Medicare (fee-for-service) Part B (Medical Insurance) beneficiaries by physicians and other healthcare professionals, aggregated by provider. Given that the data from 2020 to 2021 offers the most recent information within our study period, we selected these years for our analysis. However, we acknowledge the potential limitations on the external validity of our findings beyond this timeframe, especially considering the significant impact of the COVID-19 pandemic on physician procedural volume and compensation, as noted in the literature (13). Our study included only neurosurgeons practicing in neighborhoods historically graded by the HOLC. We identified HOLC neighborhoods using the Mapping Inequality Project Digitized Maps and ArcGIS Hub digitized overlays, following the methods similar to Deo et al. (3) and Hollenbach et al. (1, 14, 15). Figure 1 is a HOLC map of Chicago to provide an illustration of historical HOLC maps. Demographic data for each ZIP code were sourced from the U.S. Census Bureau’s ZIP Code Tabulation Areas (ZCTAs) profiles. ZCTAs are geographic units created by the Census Bureau, facilitating mapping, display, and geographic analyses based on United States Postal Service (USPS) ZIP codes.

Additionally, Area Deprivation Index (ADI) data for each ZIP code were collected from the open-access Neighborhood Atlas provided by the Center for Health Disparities Research at the University of Wisconsin (12). The ADI serves as a multifaceted tool extensively employed in numerous studies to evaluate socioeconomic disadvantage and its influence on health outcomes. Comprising 17 distinct measures, it provides a comprehensive understanding of the social determinants of health within specific areas. These measures encompass various indicators such as income, education, employment, housing quality, access to transportation, neighborhood safety, availability of healthcare services, environmental factors, food environment, social support networks, access to technology, housing affordability, language barriers, disability status, health insurance coverage, and demographic composition. By synthesizing these measures, the ADI offers a comprehensive framework for assessing socioeconomic disadvantage and its implications for health equity and well-being at the community level. A higher ADI score indicates greater levels of deprivation, rendering it a valuable tool for identifying and addressing disparities in various social and health outcomes across different regions.

### Defining exposure and end point

**Figure 2:**
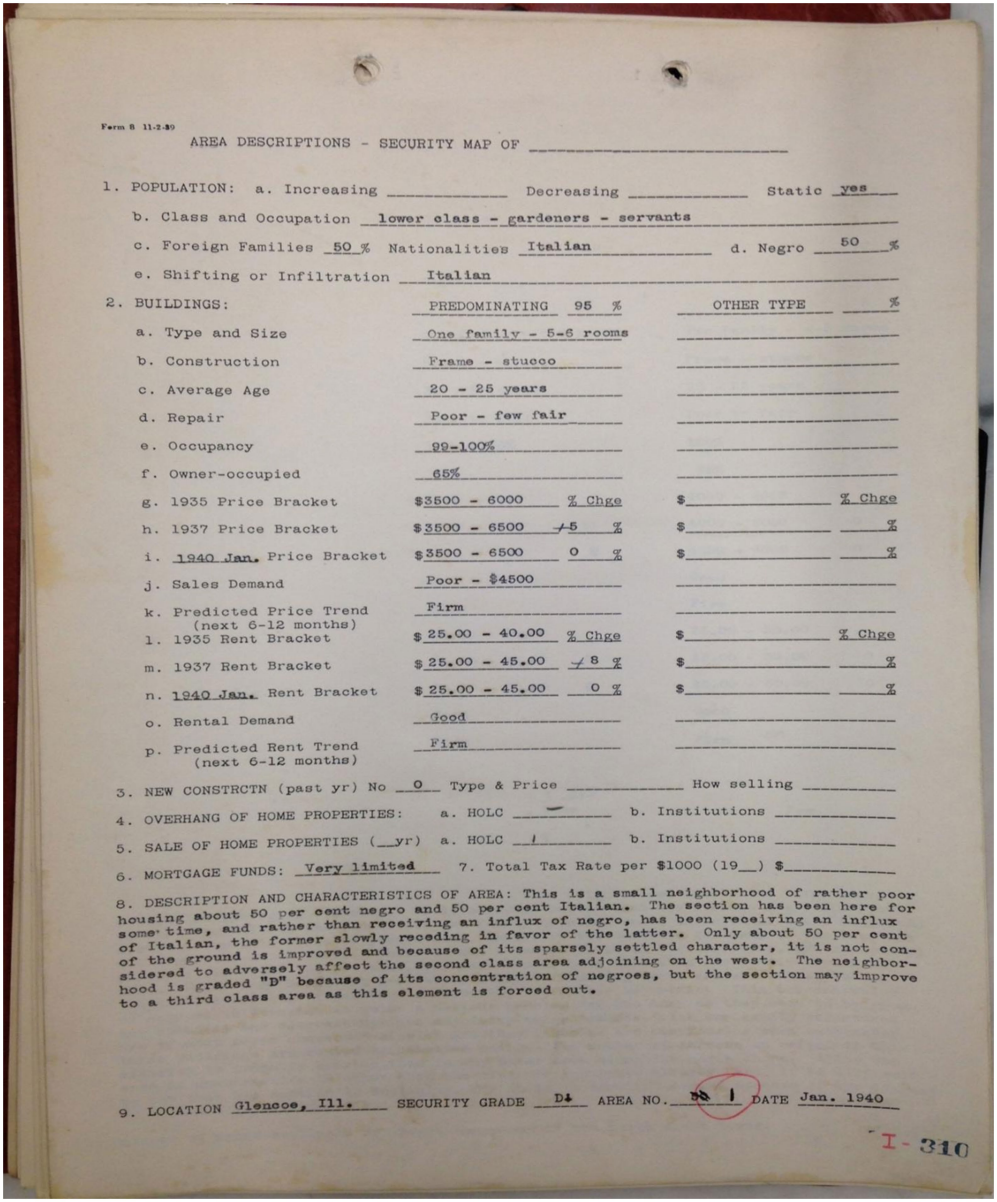
Area description of a Homeowners’ Loan Corporation (HOLC) Grade D Community: Is a historical document prepared by the HOLC of a Grade D area within Chicago. It serves as a poignant illustration of the criteria HOLC employed to categorize neighborhoods. Notably, the figure draws attention to the occupations of residents, the presence of foreign.

Each ZIP code was categorized based on an HOLC grade, assigned in the 1930s, ranging from A to D. Each grade signified unique characteristics and varying investment risk levels. Grade A neighborhoods, “Best,” were characterized by predominantly White populations, higher incomes, and stable property values. Grade B neighborhoods, “Still Desirable,” featured relatively stable communities with limited economic challenges. Grade C neighborhoods, “Definitely Declining,” encompassed a mix of working-class residents and some economic instability. Grade D neighborhoods, “Hazardous,” were least desirable, marked by lower-income populations, minority communities, or significant economic challenges. Historically, redlined HOLC Grade D neighborhoods were subject to discriminatory lending practices and substantial disinvestment, which disproportionately impacted minority populations. Today, these grades represent persistent patterns of racial and socioeconomic composition. Figure 2 provides a historical document that illustrates key criteria employed by the HOLC to categorize neighborhoods, highlighting the factors that influenced their stratification (14). The image was licensed under a Creative Commons Attribution-NonCommercial-ShareAlike 4.0 International License.

Additionally, our study identified HOLC Grade D neighborhoods that underwent gentrification, identified by their current ADI scores falling outside the fourth quartile, indicative of the highest deprivation levels. Gentrification, marked by an urban revitalization process involving an influx of more affluent residents and investments into previously deteriorated or lower-income neighborhoods, often results in escalating property values, demographic shifts, and the potential displacement of long-term, lower-income residents.

Similarly, utilizing contemporary ADI metrics, we identified neighborhoods previously classified as HOLC Grade A, B, and C that experienced de-gentrification. In our analysis, de-gentrification was delineated by HOLC Grade A neighborhoods with ADI scores falling below the top quartile, HOLC Grade B neighborhoods below the second quartile, and HOLC Grade C zones below the third quartile. De-gentrification signifies the transformation of once-affluent neighborhoods, evidenced by declining property values and a demographic shift toward lower-income or less affluent residents, often influenced by a combination of socioeconomic factors and policy alterations.

ADI has been leveraged in prior research to illustrate neighborhood change in historically redlined areas, as evidenced by previous publications (16). The ADI’s ability to offer a comprehensive assessment of the socioeconomic characteristics of neighborhoods, its capacity to capture longitudinal data for tracking changes in socioeconomic status, and its standardized and validated nature enhance its utility as a tool for comparing gentrification across diverse geographic regions and populations. By encompassing multiple dimensions of disadvantage, the ADI provides valuable insights into the complex socioeconomic dynamics driving gentrification processes.

Reimbursement values provided by the data source represent the average annual Medicare allowed amount for the services rendered. This allowed amount encompasses the portion paid by Medicare, the deductible and coinsurance for which the beneficiary is responsible, and any contributions from third-party payers. Neighborhoods were categorized into overrepresented and underrepresented groups using an adapted representativeness ratio, following the methodology outlined by Snyder et al. (17) and reflected below (17). The ratio was defined as 1.0 when the number of neurosurgeons in a HOLC neighborhood matched the proportion of residents in that neighborhood. A ratio greater than 1.0 indicates overrepresentation and a ratio less than 1.0 indicates underrepresentation.


$$\mathrm{Representation}\;\mathrm{Ratio}=\frac{\frac{\left(\mathrm{Number}\;\mathrm{Neurosurgeons}\;\mathrm{in}\;\mathrm{Zip}\;\mathrm{Code}\right)}{\left(\begin{array}{l} \mathrm{Number}\;\mathrm{of}\;\mathrm{Neurosurgeons}\;\mathrm{in}\;\mathrm{all}\;\mathrm{HOLC}\;\\ \;\mathrm{Zip}\;\mathrm{Codes} \end{array}\right)}}{\frac{\left(\mathrm{Total}\;\mathrm{Population}\;\mathrm{in}\;\mathrm{Zip}\;\mathrm{Code}\right)}{\left(\mathrm{Total}\;\mathrm{Population}\;\mathrm{in}\;\mathrm{all}\;\mathrm{HOLC}\;\mathrm{Zip}\;\mathrm{Codes}\right)}}$$

### Statistical analysis

We conducted independent sample t-tests and to assess baseline disparities among neighborhoods. HOLC Grade D neighborhoods were individually compared to HOLC Grade A, B, and C neighborhoods. We further examined the potential impact of gentrification on neurosurgeon representation by comparing gentrified Grade D areas to non-gentrified Grade D areas, as well as de-gentrified to unchanged neighborhoods.

In the first analysis, we evaluated the degree of neurosurgeon overrepresentation. We initially performed univariable logistic regression analysis to examine disparities in neurosurgeon representation, followed by multivariable regression analysis. The multivariable analysis the following included covariates ADI, racial demographics (percentages of White, Black, and Asian residents), total population, state, and calendar year. Notably, ADI was not included as a covariate in the multivariable analysis of comparing impact of gentrification and de-gentrification, as it was utilized for the stratification of these patterns.

Additionally, we compared total service costs billed and reimbursement received through univariate analysis, employing independent sample t-tests. Subsequently, multivariable analysis was conducted using analysis of covariance (ANCOVA), with covariates including calendar year, state, total count of Medicare beneficiaries who received services from the provider, and total number of services performed by the provider. All significance tests were two-sided, and statistical significance was set *a priori* at *p* < 0.05. The statistical analyses were conducted using IBM SPSS version 29.

## Results

We reviewed all 202 formerly HOLC graded cities mapped by the Mapping Inequalities Project. However, 111 were excluded from our study due to the absence of practicing neurosurgeons or incomplete data. Our analysis covered 257 neighborhoods within 91 cities across the United States. The distribution of neighborhoods by HOLC grade was as follows: 5.40% were classified as Grade A, 18.80% as Grade B, 45.8% as Grade C, and 30.00% as Grade D. Notably, a substantial proportion (79.48%) of the Grade D neighborhoods have undergone gentrification.

HOLC Grade A, B, and C neighborhoods exhibited a statistically significant higher proportion of White and Asian residents compared to Grade D neighborhoods (Table 1). Conversely, Grade D neighborhoods had a higher proportion of Black residents than Grade A, B, and C neighborhoods (*p* < 0.001 for all comparisons) (Table 1). No statistically significant differences were observed in the proportions of White (*p* = 0.092) and Black (*p* = 0.107) residents between gentrified and non-gentrified neighborhoods. Non-gentrified neighborhoods had a smaller percentage of Asian residents compared to gentrified areas (1.82% vs. 4.00%, *p* < 0.001), indicated in Table 1. Conversely, when comparing de-gentrified areas to unchanged areas, the former showed a lower proportion of White residents (56.98% vs. 62.58%, *p* = 0.003) and Asian residents (6.30% vs. 9.93%, *p* < 0.001), along with a higher proportion of Black residents (26.12% vs. 14.94%, *p* < 0.001). Table 1 summarizes baseline characteristics of the included zip codes categorized by HOLC Grade, gentrification, and de-gentrification status.

**Table 1:** Baseline neighborhood demographic characteristics.

	% White residents		% Asian residents		% Black residents		ADI	
HOLC grade	Mean	*p*-value	Mean	*p*-value	Mean	*p*-value	Mean	*p*-value
A (*n* = 14)	72.42	**<0.001**	9.96	**<0.001**	10.21	**<0.001**	18.00	**<0.001**
B (*n* = 51)	66.80	**<0.001**	11.33	**<0.001**	10.28	**<0.001**	41.70	1.000
C (*n* = 114)	57.71	**0.046**	6.98	**<0.001**	22.92	**<0.001**	49.34	**0.002**
D (*n* = 78)	54.05	Ref	2.75	Ref	36.21	Ref	39.86	Ref
**Gentrification**								
Non-gentrified neighborhoods (*n* = 16)	50.84	0.092	1.82	**<0.001**	39.11	0.107	80.54	**<0.001**
Gentrified neighborhoods (*n* = 62)	56.97		4.00		32.81		20.38	
**De-Gentrification**								
Unchanged neighborhoods (*n* = 107)	62.58	**0.003**	9.93	**<0.001**	14.94	**<0.001**	24.56	**<0.001**
De-gentrified neighborhoods (*n* = 56)	56.98		6.30		26.12		85.38	

ADI, Area Deprivation Index; HOLC, Home Owners Loan Corporation. The HOLC grade comparisons in the table compared each HOLC Grade D neighborhood to HOLC Grade A, B, and C neighborhoods individually, assessing their respective characteristics and disparities. Bold values indicate statistically significant *p*-values.

Neurosurgeons in HOLC Grade D areas cared for more Medicare beneficiaries than those in Grade A (155 vs. 122, *p* = 0.032) and Grade B (155 vs. 131, *p* = 0.013) areas. However, there was no significant difference when compared with Grade C (155 vs. 165, *p* = 0.806). Conversely, neurosurgeons in HOLC Grade D areas performed fewer services to Medicare beneficiaries than those in Grade C (386 vs. 462, *p* = 0.021). No significant differences were observed in number of services performed between Grade D and A (386 vs. 335, *p* = 1.000) or B (386 vs. 340, *p* = 0.957). Conversely, gentrified areas demonstrated a significant contrast, providing care to a higher number of Medicare beneficiaries than non-gentrified areas (164 vs. 123, *p* < 0.001), and conducting more services (408 vs. 289, *p* = 0.003). When comparing de-gentrified areas and unchanged areas, our analysis found no statistically significant difference in the number of Medicare beneficiaries (148 vs. 155, *p* = 0.279) and the total services performed (396 vs. 438, *p* = 0.168) by neurosurgeons.

In multivariable models, neurosurgeons were more likely to be overrepresented in HOLC Grade A (OR = 4.37, 95%CI: 2.08, 9.16, *p* < 0.001), B (OR = 1.99, 95%CI: 1.18, 3.38, *p* = 0.011), and C (OR = 2.37, 95%CI: 1.57, 3.59, *p* < 0.001) neighborhoods when compared to HOLC Grade D neighborhoods. Gentrified neighborhoods were twice as likely of being overrepresented compared to non-gentrified areas (OR = 2.06, 95%CI: 1.02, 4.16, *p* = 0.044). In contrast, unchanged areas exhibited a higher likelihood of being overrepresented than their de-gentrified counterparts (OR = 1.92, 95%CI: 1.27, 2.90; *p* = 0.002). Table 2 provides results of both unadjusted and adjusted models by HOLC grade, gentrification, and de-gentrification status.

**Table 2:** Neighborhood neurosurgeon representation by HOLC grade, redlined status, gentrification, and de-gentrification status.

	Univariable			Multivariable		
HOLC grade	OR	95%CI	*p*-value	OR	95%CI	*p*-value
A (*n* = 14)	1.29	0.71–2.33	0.395	4.37	2.08–9.16	**<0.001**
B (*n* = 51)	0.81	0.56–1.19	0.289	1.99	1.18–3.38	**0.011**
C (*n* = 114)	1.31	0.97–1.76	0.080	2.37	1.57–3.59	**<0.001**
D (*n* = 78)	Ref.			Ref.		
Non-gentrified neighborhoods (*n* = 16)	1.62	0.94–2.78	0.083	2.06	1.02–4.16	**0.044**
Gentrified neighborhoods (*n* = 62)	Ref.			Ref.		
Unchanged neighborhoods (*n* = 107)	1.27	0.90–1.78	0.169	1.92	1.27–2.90	**0.002**
De-gentrified neighborhoods (*n* = 56)	Ref.			Ref.		

OR, Odds Ratio; CI, Confidence Interval; HOLC, Home Owners Loan Corporation; Ref, Reference. In the multivariable model comparing HOLC Grade D to Grades A, B, and C, covariates included ADI, the percentage of residents who are Black, White, and Asian, the total population of the neighborhood, state, and calendar year. However, in the multivariable analyses examining the impact of gentrification and de-gentrification, ADI was not utilized as a covariate. Bold values indicate statistically significant *p*-values.

In adjusted models, neurosurgeons in HOLC Grade D areas on average billed significantly less compared to those in Grade A ($467,342.10 vs. $973,701.25, *p* < 0.001), B ($467,342.10 vs. $930,141.69, *p* < 0.001), and C ($467,342.10 vs. $584,081.31, *p* = 0.012) areas. A similar trend was observed in reimbursement: neurosurgeons in HOLC Grade D areas received less compared to those in Grade A ($109,163.77 vs. $142,999.88, *p* < 0.001), B ($109,163.77 vs. $131,459.02, *p* < 0.001), and C ($109,163.77 vs. $129,070.733, *p* < 0.001) regions. Likewise, neurosurgeons practicing in non-gentrified areas billed significantly lower amounts than their counterparts in gentrified areas ($459,218.34 vs. $496,855.33, *p* < 0.001). Additionally, there was a notable disparity in reimbursement amounts between the two groups ($101,149.15 vs. $106,492.74, *p* < 0.001). In de-gentrified areas, neurosurgeons billed less compared to those in unchanged areas ($572,637.12 vs. $779,971.51, *p* < 0.001). However, the difference in reimbursement between the two was not statistically significant ($132,976.05 vs. $132,173.33, *p* = 0.821). The outcomes of both unadjusted and adjusted analyses pertaining to billing and reimbursement practices are summarized in Table 3.

**Table 3:** Comparative billing and reimbursement rates for neurosurgeons across HOLC grades.

	Billing				Reimbursement			
	Univariable		Multivariable		Univariable		Multivariable	
	*M*	*p*-value	*M*	*p*-value	*M*	*p*-value	*M*	*p*-value
HOLC grade								
A (*n* = 14)	864938.22	**<0.001**	973701.25	**<0.001**	121899.51	1.000	142999.88	**<0.001**
B (*n* = 51)	839389.59	**<0.001**	930141.69	**<0.001**	113598.45	1.000	131459.02	**<0.001**
C (*n* = 114)	634089.90	**<0.001**	584081.31	**0.012**	138024.52	**<0.001**	129070.71	**<0.001**
D (*n* = 78)	458182.34	Ref.	467342.10	Ref.	109297.08	Ref.	109163.77	Ref.
**Gentrification**								
Non-gentrified neighborhoods (*n* = 16)	367932.82	**0.035**	459218.34	**<0.001**	76585.86	**<0.001**	106492.74	**<0.001**
Gentrified neighborhoods (*n* = 62)	478831.33	496855.33	112620.34	101149.15
**De-gentrification**								
Unchanged neighborhoods (*n* = 107)	793056.78	**<0.001**	779971.51	**<0.001**	134660.56	0.243	132976.05	0.821
De-gentrified neighborhoods (*n* = 56)	541124.38		572627.17		126988.04		132173.33	

M, Mean; HOLC, Home Owners Loan Corporation; Ref, References. Bold values indicate statistically significant *p*-values.

## Discussion

Our findings shed light on the enduring influence of structural racism, as exemplified by redlining, on the geographical distribution of neurosurgeons in contemporary U.S. neighborhoods. The disparities in neurosurgeon representation, as influenced by HOLC grades, gentrification, and de-gentrification status, underscore the long-lasting impact of structural racism on equitable access to specialized medical care.

Our results, which demonstrate that neighborhoods historically graded A, B, and C had a significantly higher proportion of White and Asian residents than Grade D neighborhoods, underscore the persistent impact of discriminatory housing practices on the racial composition of communities. The higher percentage of Black residents in Grade D neighborhoods highlights how structural racism, dating back to redlining, continues to perpetuate disparities that affect the most vulnerable populations. Our findings agree with studies that have shown neighborhoods historically designated as Grade D continue to exhibit a higher proportion of Black residents (6, 7). Individuals living in redlined neighborhoods were trapped in cycles of poverty and limited opportunities, unable to relocate to neighborhoods with better resources and improved socioeconomic conditions (6–8). Historical redlining contributes to the persistence of racial segregation and disparities in access to resources and opportunities in these neighborhoods (8).

Additionally, this study reveals a substantial overrepresentation of neurosurgeons in HOLC A, B, and C neighborhoods compared to Grade D, suggesting that neurosurgical services are more readily accessible in neighborhoods with a history of socioeconomic advantage. In contrast, HOLC Grade D neighborhoods, often characterized by lower-income populations and minority communities, exhibited a notable underrepresentation of neurosurgeons. This underrepresentation may indicate reduced access to neurosurgical care in historically disadvantaged neighborhoods. Numerous studies have documented persistent healthcare disparities in historical redlined areas in both access to healthcare and outcomes (1, 2, 18). Residents may experience reduced health insurance coverage and higher prevalence of chronic health conditions due to limited economic opportunities, compounding their barriers to healthcare access (1, 2). These findings underscore the enduring impact of redlining on healthcare disparities in marginalized communities.

Expanding on these, economic implications for neurosurgeons practicing in these various HOLC-graded neighborhoods further illustrate the legacy of redlining. Neurosurgeons in redlined neighborhoods billed significantly less than their counterparts in more socioeconomically advantaged Grade A, B, and C areas. The disparities were not only evident in billing practices but were also reflected in the reimbursement rates. Differences in billing and reimbursement might demonstrate several factors, including patient ability to pay, insurance coverage variations, and perhaps even differences in the complexity of procedures performed (1, 2, 19). This may underscore obstacles faced by Grade D communities, such as barriers to care, lack of transportation, longer wait times, or delayed diagnosis and care (20, 21). This financial discrepancy not only highlights economic challenges faced by healthcare professionals in these redlined areas but also emphasizes the systemic economic constraints these regions continue to grapple with (22). This financial strain could deter new practitioners from establishing practices and investing resources in Grade D areas, perpetuating the cycle of limited healthcare accessibility in these communities.

Our study provides an insightful analysis of how gentrification influences healthcare landscapes. It reveals that gentrified neighborhoods, despite not showing significant changes in racial demographics, tend to have a higher representation of neurosurgeons. This suggests that gentrification, marked by urban renewal and demographic shifts, may attract specialized medical professionals, potentially benefiting the local population. In contrast, areas experiencing de-gentrification show a decline in White and Asian populations but an increase in Black residents. These areas are less likely to have a high representation of certain medical professionals, underlining the complex dynamics of urban changes and their impact on healthcare access and equity, especially in the context of historical and ongoing structural racism.

Gentrification typically leads to increased property values and rents, impacting housing affordability. Additionally, the socio-economic changes accompanying gentrification highlight the enduring effects of historical redlining practices on access to specialized healthcare like neurosurgery. Our findings indicate that neurosurgeons are less likely to be reimbursed at the same rates in non-gentrified communities as in gentrified ones. This discrepancy could be attributed to varying socio-economic profiles of these areas, differences in insurance preferences, or a shift in the quality of care provided (22). Shifting demographics and economic opportunities hint at a redistribution of resources, which can hinder healthcare access. The rising costs associated with gentrification often lead to the displacement of long-standing, predominantly minority, residents (23, 24). Conversely, de-gentrification can exacerbate healthcare disparities by driving away essential resources, including healthcare facilities and professionals, from formerly more affluent neighborhoods. As property values drop and wealthier residents leave, the remaining community often faces deteriorating healthcare infrastructure, resulting in reduced access to quality medical services and widening healthcare delivery gaps (25).

## Limitations and future directions

Several limitations should be acknowledged when interpreting the findings of our study. Firstly, our analysis relies on historical HOLC grades assigned in the 1930s, which may not fully reflect the current socioeconomic conditions of neighborhoods. Changes in demographics and economic factors over time could have influenced the healthcare landscape in ways that were not captured by our study. Additionally, our focus on neurosurgeon representation as a proxy for healthcare access may not fully capture the broader spectrum of medical specialties and general healthcare services available in redlined neighborhoods. Future research should explore the impact of historical redlining on access to a wider range of healthcare services.

Furthermore, it’s important to note that our analysis is observational, and while we controlled for various demographic and socioeconomic factors, there may be unmeasured confounders that could influence our results. As a result, our study does not establish a causal relationship between historical redlining and neurosurgeon representation but rather identifies associations. Additionally, our analysis did not specifically account for the presence of academic institutions or trauma care centers within the CMS database, which may have implications for neurosurgeon practice patterns. Future studies should consider incorporating these factors to provide a more comprehensive understanding of healthcare access in redlined neighborhoods.

## Conclusion

This study underscores the enduring influence of historical redlining on the distribution of neurosurgeons in contemporary neighborhoods. Disparities in neurosurgeon representation based on HOLC grades, and gentrification status highlight the ongoing impact of structural racism on healthcare access. Efforts to address healthcare disparities must consider the historical roots of these inequalities and work toward equitable access to specialized medical care for all communities, particularly those with a history of socioeconomic disadvantage.

## Data availability statement

Publicly available datasets were analyzed in this study. This data can be found here: 1. https://www.neighborhoodatlas.medicine.wisc.edu 2. https://www.cms.gov/medicare/payment/fee-schedules/physician 3. https://dsl.richmond.edu/panorama/redlining/ 4. https://www.arcgis.com/home/item.html?id=ef0f926eb1b146d082c38cc35b53c947.

## Ethics statement

Ethical approval was not required for the study involving humans in accordance with the local legislation and institutional requirements. Written informed consent to participate in this study was not required from the participants or the participants’ legal guardians/next of kin in accordance with the national legislation and the institutional requirements.

## Author contributions

J-LK: Conceptualization, Data curation, Formal analysis, Investigation, Methodology, Validation, Visualization, Writing – original draft, Writing – review & editing. JD: Conceptualization, Formal analysis, Methodology, Validation, Visualization, Writing – original draft, Writing – review & editing. BJ: Conceptualization, Data curation, Formal analysis, Validation, Visualization, Writing – original draft, Writing – review & editing. AH: Conceptualization, Investigation, Methodology, Validation, Visualization, Writing – original draft, Writing – review & editing. TN-C: Conceptualization, Validation, Visualization, Writing – review & editing. DF: Conceptualization, Writing – review & editing. MB: Conceptualization, Writing – review & editing. LF: Data curation, Writing – review & editing. SE: Conceptualization, Investigation, Methodology, Project administration, Resources, Supervision, Validation, Visualization, Writing – original draft, Writing – review & editing.
