# Supplementary material for: The impact of historical redlining on neurosurgeon distribution and reimbursement in modern neighborhoods
Source: Front Public Health. 2024 May 7;12:1364323. doi: 10.3389/fpubh.2024.1364323 (PMC11106381; doi:10.3389/fpubh.2024.1364323)
Supplement: Supplementary file 1 [file Data_Sheet_1.DOCX]

STROBE Statement—checklist of items that should be included in reports of observational studies

|  | Item No. | Recommendation | Page  No. | Relevant text from manuscript |
| --- | --- | --- | --- | --- |
| **Title and abstract** | 1 | (*a*) Indicate the study’s design with a commonly used term in the title or the abstract | 2 | Study design is indicated in the Design section of the abstract - Observational study |
|  |  | (*b*) Provide in the abstract an informative and balanced summary of what was done and what was found | 2-3 | This information is stated in the study abstract (study objective described, method and results described) |
| Introduction | | | |  |
| Background/rationale | 2 | Explain the scientific background and rationale for the investigation being reported | 4-5 | Rationale and existing literature are stated in the introduction section |
| Objectives | 3 | State specific objectives, including any prespecified hypotheses | 5 | The objective of this study is to investigate the lasting impact of redlining on present-day geographic distribution of neurosurgeons in the U.S. at the neighborhood level and to elucidate its effects on Medicare reimbursement patterns. We posit that redlining, by perpetuating socioeconomic disparities and residential segregation, has had a lasting impact on the availability of specialized medical care in marginalized communities. |
| Methods | | | |  |
| Study design | 4 | Present key elements of study design early in the paper | 6-8 | Study design is stated in the first two subsections of Methods. Key elements are all described in the methods. |
| Setting | 5 | Describe the setting, locations, and relevant dates, including periods of recruitment, exposure, follow-up, and data collection | 6-8 | Setting, contexts, dates of inclusion, are fully described in the method section under “Data Sources” and “Defining Exposure and End Point” |
| Participants | 6 | (*a*) *Cohort study*—Give the eligibility criteria, and the sources and methods of selection of participants. Describe methods of follow-up  *Case-control study*—Give the eligibility criteria, and the sources and methods of case ascertainment and control selection. Give the rationale for the choice of cases and controls  *Cross-sectional study*—Give the eligibility criteria, and the sources and methods of selection of participants |  |  |
|  |  | (*b*) *Cohort study*—For matched studies, give matching criteria and number of exposed and unexposed  *Case-control study*—For matched studies, give matching criteria and the number of controls per case |  |  |
| Variables | 7 | Clearly define all outcomes, exposures, predictors, potential confounders, and effect modifiers. Give diagnostic criteria, if applicable | 7-9 |  |
| Data sources/ measurement | 8* | For each variable of interest, give sources of data and details of methods of assessment (measurement). Describe comparability of assessment methods if there is more than one group | 6-7 | Data collection and measurement was the same for all variables and is described in the methods section under “Data Sources”. |
| Bias | 9 | Describe any efforts to address potential sources of bias |  |  |
| Study size | 10 | Explain how the study size was arrived at | 6-7 | The methods section under “Data Sources” describes the inclusion of neurosurgeons practicing in neighborhoods historically graded by the HOLC. |

Continued on next page

| Quantitative variables | 11 | Explain how quantitative variables were handled in the analyses. If applicable, describe which groupings were chosen and why | 7 | HOLC grading scheme is described in the method section under “Defining Exposure and End Point” |
| --- | --- | --- | --- | --- |
| Statistical methods | 12 | (*a*) Describe all statistical methods, including those used to control for confounding | 8-9 | These are described in the method section under “Statistical Analysis”. |
|  |  | (*b*) Describe any methods used to examine subgroups and interactions | 8-9 | These are described in the method section under “Statistical Analysis”. Interaction examination was not applicable. |
|  |  | (*c*) Explain how missing data were addressed | N/A |  |
|  |  | (*d*) *Cohort study*—If applicable, explain how loss to follow-up was addressed  *Case-control study*—If applicable, explain how matching of cases and controls was addressed  *Cross-sectional study*—If applicable, describe analytical methods taking account of sampling strategy | N/A |  |
|  |  | (*e*) Describe any sensitivity analyses | N/A |  |
| Results | | | | |
| Participants | 13* | (a) Report numbers of individuals at each stage of study—eg numbers potentially eligible, examined for eligibility, confirmed eligible, included in the study, completing follow-up, and analysed | N/A | No individuals were directly involved or identifiable in the research process. |
|  |  | (b) Give reasons for non-participation at each stage | N/A |  |
|  |  | (c) Consider use of a flow diagram | N/A |  |
| Descriptive data | 14* | (a) Give characteristics of study participants (eg demographic, clinical, social) and information on exposures and potential confounders | N/A | No individuals were directly involved or identifiable in the research process. |
|  |  | (b) Indicate number of participants with missing data for each variable of interest | N/A |  |
|  |  | © *Cohort study*—Summarise follow-up time (eg, average and total amount) | N/A |  |
| Outcome data | 15* | *Cohort study*—Report numbers of outcome events or summary measures over time |  |  |
|  |  | *Case-control study—*Report numbers in each exposure category, or summary measures of exposure |  |  |
|  |  | *Cross-sectional study—*Report numbers of outcome events or summary measures |  |  |
| Main results | 16 | (*a*) Give unadjusted estimates and, if applicable, confounder-adjusted estimates and their precision (eg, 95% confidence interval). Make clear which confounders were adjusted for and why they were included | 10-12 | Justification for covariates are described in the method section under “Statistical Analysis” |
|  |  | (*b*) Report category boundaries when continuous variables were categorized |  |  |
|  |  | (*c*) If relevant, consider translating estimates of relative risk into absolute risk for a meaningful time period | N/A |  |

Continued on next page

| Other analyses | 17 | Report other analyses done—eg analyses of subgroups and interactions, and sensitivity analyses |  |  |
| --- | --- | --- | --- | --- |
| Discussion | | | | |
| Key results | 18 | Summarise key results with reference to study objectives | 13 | Key results are described at the beginning of discussion section and summarized in the conclusion. |
| Limitations | 19 | Discuss limitations of the study, taking into account sources of potential bias or imprecision. Discuss both direction and magnitude of any potential bias | 16 | Description of limitations is done under “Limitations and Future Directions” heading in the discussion |
| Interpretation | 20 | Give a cautious overall interpretation of results considering objectives, limitations, multiplicity of analyses, results from similar studies, and other relevant evidence | 13-16 | References were added and discussed where possible. Limitations were considered in the discussion. |
| Generalisability | 21 | Discuss the generalisability (external validity) of the study results | 16 | Our study focuses on neurosurgeon representation and as a specialized field, general healthcare services as well as other medical specialties may be impacted and should be investigated. |
| Other information | |  | | |
| Funding | 22 | Give the source of funding and the role of the funders for the present study and, if applicable, for the original study on which the present article is based |  |  |

*Give information separately for cases and controls in case-control studies and, if applicable, for exposed and unexposed groups in cohort and cross-sectional studies.

**Note:** An Explanation and Elaboration article discusses each checklist item and gives methodological background and published examples of transparent reporting. The STROBE checklist is best used in conjunction with this article (freely available on the Web sites of PLoS Medicine at http://www.plosmedicine.org/, Annals of Internal Medicine at http://www.annals.org/, and Epidemiology at http://www.epidem.com/). Information on the STROBE Initiative is available at www.strobe-statement.org.
